# Rab32 restriction of intracellular bacterial pathogens

**DOI:** 10.1080/21541248.2016.1219207

**Published:** 2016-09-20

**Authors:** Virtu Solano-Collado, Adam Rofe, Stefania Spanò

**Affiliations:** Institute of Medical Sciences, University of Aberdeen, Foresterhill, Aberdeen, UK

**Keywords:** bacterial pathogens, Hermansky-Pudlak syndrome, host-pathogen interaction, innate immunity, lysosome-related organelles, Rab GTPases, *Salmonella*, typhoid fever

## Abstract

Our immune system is engaged in a continuous battle against invading pathogens, many of which have evolved to survive in intracellular niches of mammalian hosts. A variety of cellular processes are involved in preventing bacterial invasion or in killing bacteria that successfully invade host cells. Recently, the Rab GTPase Rab32 emerged as critical regulator of a host defense pathway that can eliminate bacterial pathogens. *Salmonella enterica* is an intracellular bacterium and a major cause of infections and deaths in humans. Rab32 and its guanine nucleotide exchange factor BLOC-3 are essential to prevent the growth of the human-restricted *Salmonella enterica* serovar Typhi (*S.* Typhi) in mice, a non-susceptible host. The importance of the Rab32/BLOC-3 pathway has been recently confirmed by the finding that broad-host *Salmonella enterica* serovars deliver 2 bacterial effectors to neutralize this pathway and infect mice. Rab32 has also been shown to control infection by *Listeria monocytogenes*, another medically relevant intracellular pathogen. In addition, genetic evidence indicate a possible role of Rab32 in controlling leprosy, a disease caused by *Mycobacterium leprae* in humans, suggesting that a Rab32-dependent pathway can also act as a host defense pathway in humans. The Rab32 role in bacterial pathogen restriction is discussed here and compared to the function of this GTPase in other cellular processes.

## Introduction

Many cellular processes are involved in preventing bacterial pathogens from replicating within mammalian hosts (reviewed in refs. 1, 2). Phagocytic cells (i.e., macrophages and dendritic cells) are the cells of the immune system devoted to internalizing and destroying bacterial invaders. Phagocytic processes are well studied, as well as many of the processes that lead to the activation of antimicrobial mechanisms and bacterial removal.^3^ Reactive oxygen species, nitric oxide, antimicrobial peptides and toxic concentrations of heavy metals are part of the molecular arsenal used by phagocytic cells to kill bacterial pathogens.^4-8^ However, successful bacterial pathogens evolved to neutralize this antimicrobial attack and survive within phagocytic cells. Investigating the bacterial molecules involved in this host-pathogen battle has recently led to the discovery of a novel host defense pathway, where a central role is played by the Rab GTPase Rab32. This pathway protects some mammalian species, e.g. mice, from the attack of the human-adapted pathogen *Salmonella enterica* serovar Typhi (*S.* Typhi).^9,10^ However, it does not protect mice from broad-host *Salmonella enterica* (*Salmonella*) serovars, as these serovars have evolved potent bacterial effectors to neutralize this pathway.^10^ The Rab32-dependent pathway might be a broad host-defense pathway against intracellular pathogens. In fact, genetic evidence suggests a role for Rab32 in preventing *Mycobacterium leprae* infections.^11,12^ In addition, Rab32 and its close homolog Rab38 have also been reported to control *Listeria monocytogenes*.^13^ In this mini-review we discuss the findings that led to the identification of the Rab32 host defense pathway in the context of *Salmonella* infections, evidence of its involvement in controlling other intracellular pathogens and the role of Rab32 in other cellular trafficking processes.

### *Salmonella*: Establishing different diseases in different hosts

*Salmonella* is a facultative intracellular pathogen that is well adapted to live in mammalian hosts. There are more than 2 thousand serovars belonging to this species and they constitute a main cause of infectious diseases. *S.* Typhi and the less common *Salmonella enterica* serovar Paratyphi A (*S.* Paratyphi) are responsible for more than 26 million cases of typhoid fever, a life-threatening disease that kills hundreds of thousands of people each year, mainly in the developing world.^14-16^ In contrast, all the other *Salmonella* serovars cause in humans a milder and local infection, i.e. a gastroenteritis known as salmonellosis. Remarkably, while *S.* Typhi and *S.* Paratyphi are host-restricted serovars that can only infect humans, the majority of *Salmonella* serovars can infect a broad-range of animal hosts and cause, in many of these hosts, a systemic disease that is often fatal.^17^ For example, the well-known *Salmonella* serovar Typhimurium is responsible for common outbreaks of gastroenteritis in humans but can infect systemically cattle, sheep and mice, with the last being a common laboratory model of *Salmonella* systemic infection.

*Salmonella* colonizes the host by initially invading the epithelial cells lining the intestinal tract. This invasion is an active process induced by *Salmonella* via the delivery of effectors through a specialized secretion system, called type III secretion system.^18^ These effectors act coordinately to induce macropinocytosis and the formation of a compartment known as the *Salmonella*-containing vacuole, where this pathogen thrives and multiplies. Some of the type III secretion effectors also participate in inducing an inflammatory response that confers *Salmonella* an advantage over commensal intestinal bacteria.^19^ Once *Salmonella* has multiplied in the intestinal epithelial cells, the bacterium can get access to the underlying connective tissue or lamina propria.^20^ The lamina propria is rich in macrophages and other immune cells that mediate the initial immune response to pathogens. In this tissue an important battle is played at the level of the macrophages and possibly other phagocytic cells.^21^ These cells can eliminate bacteria, including many pathogens. However, if the bacteria succeed in overcoming host-cell defenses that act to restrict bacterial growth, the host will not be able to mount an immune response to the bacteria and the infection will spread systemically. Bacteria will survive and multiply in phagocytic cells, with the result that the same cells that should have restricted the infection actually become a vehicle to spread the infection to the bloodstream and peripheral organs. Depending on the specific host and the specific *Salmonella* serovar involved, either the bacterium will be efficiently killed in the intestinal lamina propria or a systemic infection will develop. The function of a second type III secretion system is required for a *Salmonella* broad-host serovar, *S.* Typhimurium, to establish a systemic infection in the mouse.^22^ This second type III secretion system is only expressed once the bacteria have established their intracellular niche, and is required for the bacteria to survive and replicate in macrophages.^23^ However, the exact mechanisms through which the effectors delivered by this system favor bacterial survival and the establishment of a systemic infection are only recently starting to emerge.

### *Salmonella* Typhi host-restriction: Critical role of a Rab32/BLOC-3-dependent trafficking pathway

*S.* Typhi can only infect humans.^16^ Although it is responsible for a life-threatening disease that has been affecting humans for centuries, the molecular mechanisms at the basis of its human-adaptation and the ability to spread systemically are still mostly unknown. For many years, the entry and trafficking of the broad-host serovar *S.* Typhimurium within infected cells, particularly epithelial cells, have been investigated in detail. *S.* Typhimurium is first internalized in an EEA1 positive compartment, which then acquires Rab7 and endolysosomal markers, such as the glycoprotein LAMP-1.^24,25^ This compartment is highly dynamic, exchanges material with the endolysosomal system and engages cytoskeleton elements (reviewed in refs. 26-28). Only recently a main difference in the intracellular biology of *S.* Typhi and *S.* Typhimurium has been reported. *S.* Typhi recruits the GTPases Rab32, Rab38 and Rab29 (also known as Rab7L1) to its vacuole, but *S.* Typhimurium does not.^9,29^ These 3 proteins constitute a subfamily of Rab GTPases distinct from the primordial endocytic and exocytic Rab subfamilies.^10,30^ Both in epithelial cells and macrophages the majority of *S.* Typhi-containing vacuoles are decorated by Rab32, Rab38 and Rab29, while these Rab GTPases are never recruited to the *S.* Typhimurium-containing vacuole.^9,29^ What is the molecular basis for this striking difference? Why does the *S.* Typhimurium vacuole not acquire Rab32, Rab38 and Rab29? The reason resides in 2 bacterial effectors that target these GTPases. They are both expressed and delivered by *S.* Typhimurium, but not *S.* Typhi. The first to be identified was GtgE, a bacteriophage protein previously shown to be required for *S.* Typhimurium virulence in mice.^29,31^ GtgE is a cysteine protease that specifically cleaves these 3 related Rab GTPases.^9,29,32^ The second *S.* Typhimurium effector targeting Rab32 is SopD2, a protein that acts as a GTPase activating protein (GAP) for Rab32 and induces its dissociation from the vacuolar membrane.^10^

Bacterial effectors and toxins have very often worked as excellent tools to clarify fundamental processes in cell biology. One of the most paradigmatic examples is the *Listeria monocytogenes* effector ActA that has greatly contributed to the discovery of the molecular basis of actin nucleation.^33^ Likewise, the *S.* Typhimurium effector GtgE played a critical role in the identification of a novel cell biology process, i.e., a trafficking pathway controlling bacterial growth. GtgE was initially described as a bacterial effector preventing Rab29 localization onto the *S.* Typhimurium-containing vacuole. It was then shown to be a cysteine protease highly specific for Rab29, Rab32 and Rab38.^9,29^ Based on the observation that GtgE is conserved in the majority of broad-host *Salmonella* serovars and absent from *S.* Typhi and *S.* Paratyphi, the hypothesis was made that it confers *Salmonella* serovars the ability to infect a broad-range of hosts. This hypothesis was initially tested in primary murine macrophages, which are able to clear the human-restricted *S.* Typhi and prevent *S.* Typhi from infecting mice. When GtgE was introduced in the *S.* Typhi genome, macrophages were no longer able to kill *S.* Typhi.^9^ In addition, mice inoculated with a strain of *S.* Typhi expressing GtgE were systemically infected with this strain. This ability of GtgE to enhance *S.* Typhi survival in mouse macrophages and infectivity in mice was shown to be the result of its protease activity toward Rab32. In fact, murine macrophages depleted of Rab32 were found to be unable to clear *S.* Typhi. Rab38 and Rab29 are also substrates of GtgE, but siRNA-mediated depletion of these 2 Rabs has no effect on the ability of macrophages to kill *S.* Typhi.^9^ Rab38 is highly related to Rab32 and has been shown to work redundantly with Rab32 in other cellular processes. However, as Rab38 expression is limited to specific tissues,^34^ it is probably not expressed in primary murine macrophages.

Over the past decade Rab32 and Rab38 have emerged as critical molecules in the biogenesis of lysosomal related organelles (LROs), as discussed below. The Biogenesis of Lysosome-related Organelle Complexes (BLOC) 1, 2, and 3 are also fundamental players in the biogenesis and maturation of these organelles.^35,36^ In addition to Rab32, one of these complexes, BLOC-3, was shown to be required for *S.* Typhi killing in primary murine macrophages. Interestingly, at the same time BLOC-3 was reported to be the guanine nucleotide exchange factor for Rab32 and Rab38.^37^ All these observations indicated the existence of a Rab32 regulated trafficking event controlling the killing of *S.* Typhi in primary murine macrophages. Noteworthy, mice defective for BLOC-3 were also found to be more susceptible to *S.* Typhi infection.^10^ Indeed, while wild-type mice completely clear *S.* Typhi infection, *S.* Typhi can be recovered from tissues of BLOC-3-deficient mice 2 months after the initial inoculation. These results indicate that the Rab32/BLOC-3 trafficking pathway is central for *S.* Typhi-host restriction.

### The Rab32/BLOC-3 trafficking pathway is neutralized by broad-host *Salmonella* serovars

**Figure 1.**
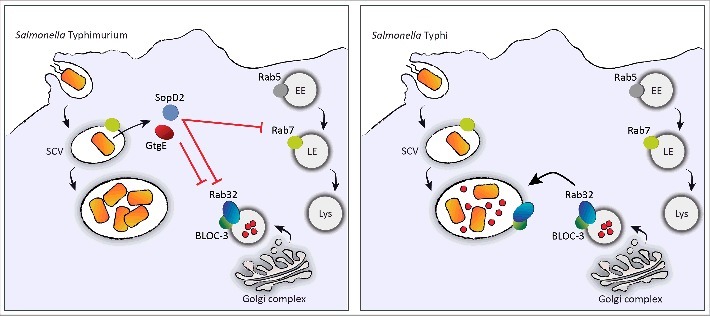
Rab32/BLOC-3 dependent antimicrobial pathway and the mechanisms used by *S*. Typhimurium to neutralize it. After entry into a host cell, *Salmonella* resides in a compartment known as the *Salmonella*-containing vacuole. In contrast to *S*. Typhi (right panel), *S*. Typhimurium (left panel) delivers to the host 2 effector proteins, GtgE and SopD2. Both effectors block the Rab32/BLOC-3-dependent antimicrobial pathway by targeting Rab32. This pathway is envisioned to deliver an antimicrobial factor (small red circles) to the *S*. Typhi-containing vacuole. GtgE is a protease that cleaves Rab32, whereas SopD2 acts as a GTPase activating protein for Rab32 and promotes its dissociation from the vacuole. In addition, SopD2 interacts with Rab7 and inhibits its activity, which is required for the delivery of endosomal contents to lysosomes. EE, early endosome; LE, late endosome; Lys, lysosome.

Does the Rab32/BLOC-3 trafficking pathway only restrict *S.* Typhi infections? Interestingly, both BLOC-3-deficient mice and Rab32-deficient mice are significantly more susceptible to the broad-host *Salmonella* serovar *S.* Typhimurium.^10^ Surprisingly, removing GtgE from *S.* Typhimurium does not significantly impair the ability of *S.* Typhimurium to infect mice and does not restore Rab32 on the *Salmonella*-containing vacuole, suggesting that *S.* Typhimurium also inhibits the recruitment of Rab32 to the bacterial vacuole through a different effector.^10^ This *S.* Typhimurium effector was identified as SopD2, an effector that is not expressed by *S.* Typhi due to the fact that it is present as a pseudogene in the *S.* Typhi genome. SopD2 is a GTPase activating protein (GAP) that inactivates Rab32.^10^ As such, it prevents Rab32-dependent trafficking in *S.* Typhimurium-infected cells and confers this broad-host *Salmonella* serovar the ability to infect mice (Fig. 1). Remarkably, deletions of both GtgE and SopD2 from the *S.* Typhimurium genome render *S.* Typhimurium unable to infect mice.^10^ SopD2 has also been recently shown to inhibit another trafficking process, the delivery of endocytic cargo to lysosomes. SopD2 acts as an inhibitor of the GDP-GTP exchange on Rab7 through direct binding of Rab7.^38^ The N-terminus of SopD2 is sufficient for Rab7 binding and inhibition of Rab7 function,^38^ while a C-terminal arginine (R315) is required for the SopD2 GAP activity on Rab32.^10^ These observations indicate that SopD2 has 2 different functions that can be assigned to different components of its structure.

Either the protease GtgE or the GAP SopD2 are able to prevent most of the Rab32 delivery to the vacuole and confer *S.* Typhimurium most of the ability to infect mice.^10^ Therefore, these effectors act in effect redundantly to block Rab32 trafficking and facilitate the establishment of a systemic infection in the mouse. Interestingly, a *S.* Typhimurium mutant defective for GtgE and SopD2, although unable to infect wild-type mice, can establish a systemic infection in mice deficient for the Rab32/BLOC-3-dependent pathway, indicating that GtgE and SopD2 are completely dispensable in the absence of this antimicrobial pathway.^10^ These recent results confirmed a central role for the Rab32/BLOC-3 trafficking pathway in host defense.

### Rab32 and related GTPases in controlling other intracellular bacterial pathogens

**Figure 2.**
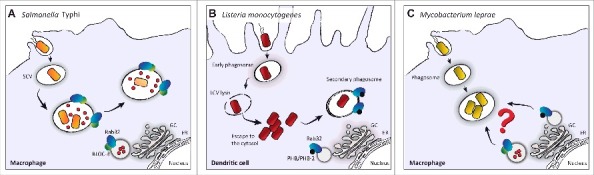
Rab32 in the control of intracellular bacterial pathogens. (A) After internalization into host macrophages, Rab32 is recruited to the *Salmonella*-containing vacuole (SCV). Rab32 and BLOC-3 are required for *S.* Typhi killing in mouse macrophages, which possibly occurs through the delivery of an antimicrobial cargo (red circles) to the bacterial vacuole.^9^ (B) After internalization in dendritic cells, *L. monocytogenes* initially resides in an early phagosome. To avoid phagolysosome formation and therefore bacterial killing, *L. monocytogenes* lyses the vacuole (*Listeria*-containing vacuole; LCV) and escapes to the cytosol where it replicates. After escaping to the cytosol, *L. monocytogenes* is captured in a membranous compartment, a multi-layered structure decorated by a complex containing Rab32 and its interactors PHB and PHB-2.^13^ (C) *M. leprae* replicates within macrophages.^71^ Although a polymorphism in Rab32 has been associated to increased susceptibility to leprosy,^11,44^ the exact role that Rab32 may play in controlling *M. leprae* infection is not known. GC, Golgi Complex; ER, Endoplasmic Reticulum.

Is the Rab32/BLOC-3-dependent host-defense pathway also able to restrict other intracellular pathogens? The genus *Mycobacterium* contains a number of species that cause serious diseases in humans, such as tuberculosis and leprosy. After internalization into host macrophages *Mycobacteria* reside in a compartment (phagosome) where they establish a suitable niche for survival.^3,39^ *Mycobacterium leprae* (*M. leprae*) is a human-adapted pathogen that causes a chronic infectious disease called leprosy.^40,41^ Most individuals exposed to *M. leprae* do not develop the disease, which may be explained, at least in part, by innate resistance provided by an individual's genetic background. Several studies have demonstrated a link between host genetic factors and susceptibility to leprosy (for reviews see refs. 42, 43). Genome-wide association studies of leprosy have been critical to identify loci associated with susceptibility to *M. leprae.*^11,12,44^ Most of the identified risk loci encode factors involved in immune responses against infections. Remarkably, a polymorphism in the Rab GTPase Rab32 was also associated with increased susceptibility to *M. leprae.*^11,12^ There is a significant lack of knowledge on how eukaryotic cells deal with *M. leprae* infections at a molecular level. The challenge of cultivating *M. leprae* outside its host together with the limited availability of suitable animal models for the study of leprosy have limited research on the mechanisms at the basis of this disease. However, the emerging importance of Rab32 trafficking pathway in the cell defense response against bacterial pathogens strongly suggests that this Rab protein may play similar roles in controlling *M. leprae* infections (Fig. 2).

In contrast to *M. leprae*, the interaction of the related pathogen *Mycobacterium tuberculosis* (*M. tuberculosis*) with the host has been investigated at cellular level and several Rab GTPases have been implicated in the interaction. At an early stage, the *M. tuberculosis* phagosome lacks antimicrobial properties. The capacity to kill pathogens is acquired during a process of phagosome maturation, which includes phagosome acidification and acquisition of oxidative and hydrolytic enzymes.^3^ This process requires the interaction of the phagosome with components of the endocytic pathway, including Rab5 and Rab7.^39^ While Rab5 associates with early phagosomes, Rab7 characterizes late phagosomes and promotes their fusion with lysosomes. *M. tuberculosis* controls the recruitment of different Rab GTPases to the phagosome during the time of infection maintaining a niche with features of an early endocytic compartment.^45^ In addition to Rab5, the *M. tuberculosis* phagosome also recruits Rab14 and Rab22a, both contributing to the maintenance of phagosomes in an early stage of maturation.^46-48^ On the contrary, the recruitment of Rab GTPases related with latter steps of phagosomal maturation is prevented. Indeed, Rab7 and Rab20, which are involved in phagosome acidification,^45^ as well as Rab10, which is involved in early stages of maturation,^49^ are not observed on the *M. tuberculosis* phagosome. Therefore, a series of Rab GTPases regulated events avoid phagosome maturation and maintain an appropriate niche for *M. tuberculosis* survival. A possible role of the Rab32/BLOC-3 pathway in the control of *M. tuberculosis* survival is still unknown.

*Listeria monocytogenes* (*L. monocytogenes*) is an intracellular pathogen that causes listeriosis, one of the most lethal foodborne infections, with 20 to 30% of clinical infections resulting in death.^50^ The bacterium is internalized into phagocytic cells and non-phagocytic host cell through receptor-mediated recognition of specific bacterial proteins.^51^ Once internalized, *L. monocytogenes* actively escapes from the bacterial vacuole into the cytosol and then is transmitted to neighboring cells through actin-based movements induced by ActA-driven actin polymerization. *L. monocytogenes* killing in dendritic cells has been recently shown to be dependent on Rab32 and Rab38.^13^ The authors showed that the Rab32-positive compartment containing *L. monocytogenes* in dendritic cells is surrounded by a multilayered membrane. They also reported that Rab32 is recruited to the surface of bacteria that have escaped to the cytosol, suggesting that this Rab32-positive compartment containing *L. monocytogenes* is a compartment sequestering bacteria from the cytosol after their initial attempt of escape from the vacuole (Fig. 2).^13^ However, they also showed that the Rab32-dependent control of *L. monocytogenes* growth is not mediated by autophagic mechanisms.^13^ Further investigations will be required to understand the exact mechanism of Rab32-mediated pathogen killing. Valuable hints could derive from the analysis of Rab32 trafficking function in non-phagocytic cells.

### Rab32 subfamily of GTPases as regulators of membrane trafficking to lysosome-related organelles (LROs)

Rab32 and its closely related isoform Rab38 have both been described as regulators of membrane trafficking to lysosome-related organelles (LROs). Rab38 expression is mostly confined to pigment cells. Rab32 is highly expressed in a variety of tissues including the liver, heart, spleen and testis,^52,53^ suggesting it performs a key role in these tissues. Expression levels are high in cells containing LROs.^29,30^ LROs are cell-type specific vesicles that share many similarities with lysosomes, yet perform a diverse range of functions. These include melanosomes,^54^ platelet granules,^55^ lytic granules,^56^ the MHC class II compartment and lamellar bodies.^57^ The observation that *S.* Typhi and *L. monocytogenes* recruit Rab32 to their vacuoles suggests that the *S.* Typhi- and the *Listeria*-containing vacuoles are also LROs. Many LROs have a critical function in immune defenses.^58,59^ Some of them, such as the lytic granules of T cells or the neutrophils granules, have a secretory function and they have also been called “secretory lysosomes.” Lytic granules contain molecules, such as perforin and granzymes, that, when released extracellularly, mediate target cell death.^59^ The neutrophils granules are filled with a variety of hydrolytic enzymes and antimicrobial peptides that are able to kill bacteria once secreted into the extracellular environment.^60^ Given the similarities between LROs and lysosomes and the fact that these 2 organelles exist within the same cell in many cell types, there must be mechanisms to distinguish between them and ensure the correct membrane proteins and luminal contents are appropriately trafficked to their target organelle.

Some genetic conditions result in deficiencies of LROs.^61^ Hermansky-Pudlack syndrome (HPS) is a genetic disorder that results in hypopigmentation, prolonged bleeding and pulmonary fibrosis, caused by defects in the biogenesis of melanosomes, platelets and lung fibroblast lamellar bodies, respectively.^62,63^ HPS is associated with mutations in BLOC-1, BLOC-2, BLOC-3 and AP-3. Rodent models of HPS display pigmentation defects (Swank et al., 1998). Interestingly, *chocolate* mice, which have a mutation in Rab38, display a mild pigmentation phenotype.^64^ This is in stark contrast to the severe hypopigmentation found when melanocytes from *chocolate* mice were depleted of Rab32 by siRNA treatment.^65^ Recently, the contribution of these Rabs in melanosome biogenesis has been explored in greater detail. Rab32 and Rab38 function within a trafficking pathway that transports melanin-producing enzymes to maturing melanosomes and interacts with components of ubiquitous trafficking machineries, such as BLOC-2, AP-1 and AP-3.^34,63,66^ Membrane-bound Rab32 and Rab38 were shown to physically interact with these proteins and this interaction was enhanced between the active GTP-bound forms of the Rabs and BLOC-2 and AP-3. This is consistent with an interaction between these proteins and the active membrane bound forms of Rab32 and Rab38.^66^

Platelets are anucleated blood cells that play an essential role in homeostasis and host defense against pathogens. Platelets contain 2 types of LROs, α-granules (AGs) and dense granules (DGs), which are released at the site of vascular injury.^67^ It has been shown that DGs originate from late endocytic organelles (possible MVBs) in a model cell line and that the sorting signals that are required to send cargo to DGs are recognized by AP-3.^68^ These cells also contain conventional lysosomes suggesting mechanisms must exist to target molecules to AGs and DGs and away from other endocytic organelles. Both Rab32 and Rab38 colocalise with AP-3 and are enriched on immature DGs. Moreover, both Rab32 and Rab38 colocalise with Rab7, but not Rab5, reinforcing the hypothesis that DGs have a late endocytic origin. Both Rab32 and Rab38 are involved in the transport of cargo-containing vesicles to DGs but their exact role in vesicle tethering or fusion has not been separated. However, siRNA depletion of either Rab32 or Rab38 cannot be fully compensated by the remaining Rab GTPase suggesting they may fulfil separate functions in platelet granule maturation.^68^

Rab32 appears to be a multifunctional protein, depending upon its cellular localization and the cell type. Rab32 has been shown to function as an A-kinase anchoring protein (AKAP) at both mitochondria and LROs.^69,70^ AKAPs act to retain other signaling enzymes at specific intracellular locations in order to coordinate protein phosphorylation events and direct them toward their substrates. Rab32 and Rab38 have been demonstrated to function within trafficking pathways for the delivery of proteins to both dense granules and melanosomes, in platelets and melanocytes, respectively. These specialized trafficking pathways employ unique trafficking machineries, including AP-3, BLOC-1, BLOC-2 and BLOC- 3. This pathway is distinct from that of conventional lysosome biogenesis. As such, it would not be surprising to find that Rab32 (and Rab38) had a role in the biogenesis of other LROs, such as the lamellar bodies of lung type II epithelial cells. However, this remains to be seen.

## Conclusions

Rab32 and its closely related homolog Rab38 control the delivery of specialized cargo to LROs. They are also recruited to the vacuoles containing bacterial pathogens, such as *S.* Typhi and *L. monocytogenes*, and they are essential to protect mammalian hosts, e.g., mice, from these pathogens. Rab32 and its GEF BLOC-3 are fundamental to restrict *S.* Typhi in mouse and are components of a powerful host defense pathway. A bacterial pathogen that can successfully infect mice, such as *S.* Typhimurium, has evolved 2 bacterial effectors to target and neutralize this pathway. The exact mechanism underlying the Rab32/BLOC-3-dependent clearance of bacterial pathogens is still unknown. However, based on the fact that Rab32 and the related Rab38 control the delivery of specialized cargo to LROs in melanocytes and platelets, it is very likely that this pathway delivers a cargo with antimicrobial properties to the *S.* Typhi- and the *L. monocytogenes*-containing vacuole. Future work clarifying the nature of this cargo will have a tremendous impact in the understanding of innate immune mechanisms protecting against bacterial pathogens.

## References

[1] Reddick L, Alto N Mol Cell 2014; 54:321-8; PMID:24766896; https://doi.org/10.1016/j.molcel.2014.03.010 24766896 PMC4023866

[2] Weiss G, Schaible UE Immunol Rev 2015; 264:182-203; PMID:25703560; https://doi.org/10.1111/imr.12266 25703560 PMC4368383

[3] Flannagan RS, Cosio G, Grinstein S Nat Rev Microbiol 2009; 7:355-66; PMID:19369951; https://doi.org/10.1038/nrmicro2128 19369951

[4] Fang FC MBio 2011; 2:2011; PMID:21896680; https://doi.org/10.1128/mBio.00141-11 21896680 PMC3171981

[5] Slauch JM Mol Microbiol 2011; 80:580-3; PMID:21375590; https://doi.org/10.1111/j.1365-2958.2011.07612.x 21375590 PMC3109634

[6] Imlay JA Nat Rev Microbiol 2013; 11:443-54; PMID:23712352; https://doi.org/10.1038/nrmicro3032 23712352 PMC4018742

[7] LaRock CN, Nizet V Biochim Biophys Acta 2015; 1848:3047-54; PMID:25701232; https://doi.org/10.1016/j.bbamem.2015.02.010 25701232 PMC4539303

[8] Neyrolles O, Wolschendorf F, Mitra A, Niederweis M Immunol Rev 2015; 264:249-63; PMID:25703564; https://doi.org/10.1111/imr.12265 25703564 PMC4521620

[9] Span∫ S, Galán JE Science 2012; 338:960-3; https://doi.org/10.1126/science.1229224 23162001 PMC3693731

[10] Span∫ S, Gao X, Hannemann S., Lara-Tejero M, Galán JE Cell. Host Microbe 2016; 19:216-26; https://doi.org/10.1016/j.chom.2016.01.004 26867180 PMC4854434

[11] Liu H. et al. Nat Genet 2015.

[12] Zhang F, Liu H, Chen S, Low H, Sun L, Cui Y, Chu T, Li Y, Fu X, Yu Y, et al. Nat Genetics 2011; 43:1247-51; PMID:22019778; https://doi.org/10.1038/ng.973 22019778

[13] Li Y, Wang Y, Zou L, Tang X, Yang Y, Ma L, Jia Q, Ni Q, Liu S, Tang L, et al. Immunity 2016; 44:422-37; PMID:26885862; https://doi.org/10.1016/j.immuni.2016.01.027 26885862

[14] Waddington CS, Darton TC, Pollard AJ J Infect 2014; 68 Suppl 1:S38-50; PMID:24119827; https://doi.org/10.1016/j.jinf.2013.09.013 24119827

[15] Kirk MD, Pires SM, Black RE, Caipo M, Crump JA, Devleesschauwer B, Döpfer D, Fazil A, Fischer-Walker CL, Hald T, et al. PLoS Med 2015; 12:e1001921; PMID:26633831; https://doi.org/10.1371/journal.pmed.1001921 26633831 PMC4668831

[16] Dougan G, Baker S Annu Rev Microbiol 2014; 68:317-36; PMID:25208300; https://doi.org/10.1146/annurev-micro-091313-103739 25208300

[17] Stevens MP, Humphrey TJ, Maskell DJ Philos Trans R Soc Lond B Biol Sci 2009; 364:2709-23; PMID:19687040; https://doi.org/10.1098/rstb.2009.0094 19687040 PMC2865095

[18] Galán JE Annu Rev Cell Dev Biol 2001; 17:53-86; https://doi.org/10.1146/annurev.cellbio.17.1.53 11687484

[19] Behnsen J, Perez-Lopez A, Nuccio SP, Raffatellu M Trends Immunol 2015; 36:112-20; PMID:25582038; https://doi.org/10.1016/j.it.2014.12.003 25582038 PMC4323876

[20] Muller AJ, Kaiser P, Dittmar KE, Weber TC, Haueter S, Endt K, Songhet P, Zellweger C, Kremer M, Fehling HJ, et al. Cell Host Microbe 2012; 11:19-32; PMID:22264510; https://doi.org/10.1016/j.chom.2011.11.013 22264510

[21] Monack DM, Mueller A, Falkow S Nat Rev Microbiol 2004; 2:747-65; PMID:15372085; https://doi.org/10.1038/nrmicro955 15372085

[22] Figueira R, Holden DW Microbiol 2012; 158:1147-61; PMID:22422755; https://doi.org/10.1099/mic.0.058115-0 22422755

[23] LaRock DL, Chaudhary A, Miller SI Nat Rev Microbiol 2015; 13:191-205; PMID:25749450; https://doi.org/10.1038/nrmicro3420 25749450 PMC5074537

[24] Steele-Mortimer O, Meresse S, Gorvel JP, Toh BH, Finlay BB Cell. Microbiol 1999; 1:33-49; PMID:11207539; https://doi.org/10.1046/j.1462-5822.1999.00003.x 11207539

[25] Meresse S, Steele-Mortimer O, Finlay BB, Gorvel JP Embo J 1999; 18:4394-403; PMID:10449405; https://doi.org/10.1093/emboj/18.16.4394 10449405 PMC1171514

[26] Bakowski MA, Braun V, Brumell JH Traffic 2008; 9:2022-31; PMID:18778407; https://doi.org/10.1111/j.1600-0854.2008.00827.x 18778407

[27] Steele-Mortimer O Curr Opin Microbiol 2008; 11:38-45; PMID:18304858; https://doi.org/10.1016/j.mib.2008.01.002 18304858 PMC2577838

[28] Liss V, Hensel M Cell Microbiol 2015; 17, 639-47; PMID:25802001; https://doi.org/10.1111/cmi.12441 25802001

[29] Span∫ S, Liu X, Galán JE Proc Natl Acad Sci USA. 2011; 108:18418-23; https://doi.org/10.1073/pnas.1111959108 22042847 PMC3215007

[30] Diekmann Y, Seixas E, Gouw M, Tavares-Cadete F, Seabra MC, Pereira-Leal JB PLoS Comput Biol 2011; 7:e1002217; PMID:22022256; https://doi.org/10.1371/journal.pcbi.1002217 22022256 PMC3192815

[31] Ho TD, Figueroa-Bossi N, Wang M, Uzzau S, Bossi L, Slauch JM J Bacteriol 2002; 184:5234-9; PMID:12218008; https://doi.org/10.1128/JB.184.19.5234-5239.2002 12218008 PMC135366

[32] Kohler AC, Span∫ S, Galán JE, Stebbins CE Acta Crystallogr D Biol Crystallogr 2014; 70:384-91; PMID:24531472; https://doi.org/10.1107/S1399004713028393 24531472 PMC3940199

[33] Kocks C, Gouin E, Tabouret M, Berche P, Ohayon H, Cossart P Cell 1992; 68:521-31; PMID:1739966; https://doi.org/10.1016/0092-8674(92)90188-I 1739966

[34] Raposo G, Marks MS, Cutler DF Curr Opin Cell Biol 2007; 19:394-401; PMID:17628466; https://doi.org/10.1016/j.ceb.2007.05.001 17628466 PMC2782641

[35] Di Pietro SM, Dell'Angelica EC Traffic 2005; 6:525-33; PMID:15941404 15941404 10.1111/j.1600-0854.2005.00299.x

[36] Dell'Angelica EC Curr Opin Cell Biol 2004; 16:458-64; PMID:15261680; https://doi.org/10.1016/j.ceb.2004.05.001 15261680

[37] Gerondopoulos A, Langemeyer L, Liang JR, Linford A, Barr FA Curr Biol 2012; 22:2135-9; PMID:23084991; https://doi.org/10.1016/j.cub.2012.09.020 23084991 PMC3502862

[38] D'Costa VM, Braun V, Landekic M, Shi R, Proteau A, McDonald L, Cygler M, Grinstein S, Brumell JH Cell Rep 2015; 12:1508-18; PMID:26299973; https://doi.org/10.1016/j.celrep.2015.07.063 26299973

[39] Fairn GD, Grinstein S Trends Immunol 2012; 33:397-405; PMID:22560866; https://doi.org/10.1016/j.it.2012.03.003 22560866

[40] Pinheiro RO, de Souza Salles J, Sarno EN, Sampaio EP Future Microbiol 2011; 6:217-30; PMID:21366421; https://doi.org/10.2217/fmb.10.173 21366421 PMC3123826

[41] Singh P, Cole ST Future Microbiol 2011; 6:57-71; PMID:21162636; https://doi.org/10.2217/fmb.10.153 21162636 PMC3076554

[42] Mazini PS, Alves HV, Reis PG, Lopes AP, Sell AM, Santos-Rosa M, Visentainer JE, Rodrigues-Santos P Front Immunol 2016; 6:658; PMID:26793196; https://doi.org/10.3389/fimmu.2015.00658 26793196 PMC4709443

[43] Fernando SL, Britton WJ Immunol Cell Biol 2006; 84:125-37; PMID:16519730; https://doi.org/10.1111/j.1440-1711.2006.01420.x 16519730

[44] Zhang FR, Huang W, Chen SM, Sun LD, Liu H, Li Y, Cui Y, Yan XX, Yang HT, Yang RD, et al. N Engl J Med 2009; 361:2609-18; PMID:20018961; https://doi.org/10.1056/NEJMoa0903753. 20018961

[45] Seto S, Tsujimura K, Koide Y Traffic 2011; 12:407-20; PMID:21255211; https://doi.org/10.1111/j.1600-0854.2011.01165.x 21255211

[46] Fratti RA, Backer JM, Gruenberg J, Corvera S, Deretic V J Cell Biol 2001; 154:631-44; PMID:11489920; https://doi.org/10.1083/jcb.200106049 11489920 PMC2196432

[47] Kyei GB, Vergne I, Chua J, Roberts E, Harris J, Junutula JR, Deretic V EMBO J 2006; 25:5250-9; PMID:17082769; https://doi.org/10.1038/sj.emboj.7601407 17082769 PMC1636625

[48] Roberts EA, Chua J, Kyei GB, Deretic V J Cell Biol 2006; 174:923-9; PMID:16982798; https://doi.org/10.1083/jcb.200603026 16982798 PMC2064384

[49] Cardoso CM, Jordao L, Vieira OV Traffic 2010; 11:221-35; PMID:20028485; https://doi.org/10.1111/j.1600-0854.2009.01013.x 20028485

[50] Swaminathan B, Gerner-Smidt P Microbes Infect 2007; 9:1236-43; PMID:17720602; https://doi.org/10.1016/j.micinf.2007.05.011 17720602

[51] Cossart P Proc Natl Acad Sci USA. 2011; 108:19484-91; PMID:22114192; https://doi.org/10.1073/pnas.1112371108 22114192 PMC3241796

[52] Bao X, Faris AE, Jang EK, Haslam RJ Eur J Biochem / FEBS 2002; 269:259-71; PMID:11784320; https://doi.org/10.1046/j.0014-2956.2001.02645.x 11784320

[53] Cohen-Solal KA, Sood R, Marin Y, Crespo-Carbone SM, Sinsimer D, Martino JJ, Robbins C, Makalowska I, Trent J, Chen S Biochimica Et Biophysica Acta 2003; 1651:68-75; PMID:14499590; https://doi.org/10.1016/S1570-9639(03)00236-X 14499590

[54] Orlow SJ 1995; 105:3-7; PMID:7615972. 10.1111/1523-1747.ep12312291 7615972

[55] McNicol A, Israels SJ 1999; 95:1-18; PMID:10403682 10403682 10.1016/s0049-3848(99)00015-8

[56] Griffiths GM, Argon Y 1995; 198:39-58; PMID:7774282 7774282 10.1007/978-3-642-79414-8_3

[57] Weaver TE, Na CL, Stahlman M 2002; 13:263-70; PMID:12243725 12243725 10.1016/s1084952102000551

[58] Huizing M, Helip-Wooley A, Westbroek W, Gunay-Aygun M, Gahl WA Annu Rev Genomics Hum Genet 2008; 9:359-86; PMID:18544035; https://doi.org/10.1146/annurev.genom.9.081307.164303 18544035 PMC2755194

[59] Holt OJ, Gallo F, Griffiths GM J Biochem 2006; 140:7-12; PMID:16877763; https://doi.org/10.1093/jb/mvj126 16877763

[60] Amulic B, Cazalet C, Hayes GL, Metzler KD, Zychlinsky A Annu Rev Immunol 2012; 30:459-89; PMID:22224774; https://doi.org/10.1146/annurev-immunol-020711-074942 22224774

[61] Wei AH, Li W Pigment Cell Melanoma Res 2013; 26:176-92; PMID:23171219; https://doi.org/10.1111/pcmr.12051 23171219

[62] Seward SL Jr, Gahl WA 2013; 132:153-60; PMID:23753089 23753089 10.1542/peds.2012-4003

[63] Di Pietro SM, Dell'Angelica EC 2005; 6:525-33; PMID:15941404 15941404 10.1111/j.1600-0854.2005.00299.x

[64] Loftus SK, Larson DM, Baxter LL, Antonellis A, Chen Y, Wu X, Jiang Y, Bittner M, Hammer JA 3rd, Pavan WJ Proc Natl Acad Sci USA 2002; 99:4471-6; PMID:11917121; https://doi.org/10.1073/pnas.072087599 11917121 PMC123672

[65] Wasmeier C, Romao M, Plowright L, Bennett DC, Raposo G, Seabra MC J Cell Biol 2006; 175:271-81; PMID:17043139; https://doi.org/10.1083/jcb.200606050 17043139 PMC2064568

[66] Bultema JJ, Ambrosio AL, Burek CL, Di Pietro SM J Biol Chem 2012; 287:19550-63; PMID:22511774; https://doi.org/10.1074/jbc.M112.351908 22511774 PMC3365991

[67] Semple JW, Italiano JE Jr, Freedman J 2011; 11:264-74; PMID:21436837 21436837 10.1038/nri2956

[68] Ambrosio AL, Boyle JA, Di Pietro SM Blood 2012; 120:4072-81; PMID:22927249; https://doi.org/10.1182/blood-2012-04-420745 22927249 PMC3496959

[69] Alto NM, Soderling J, Scott JD 2002; 158:659-68; PMID:12186851 12186851 10.1083/jcb.200204081 PMC2174006

[70] Park M, Serpinskaya AS, Papalopulu N, Gelfand VI Curr Biol 2007; 17:2030-4; PMID:17997311; https://doi.org/10.1016/j.cub.2007.10.051 17997311 PMC2330279

[71] Sibley LD, Franzblau SG, Krahenbuhl JL Infect Immun 1987; 55:680-5; PMID:3546136 3546136 10.1128/iai.55.3.680-685.1987 PMC260393

